# Vertical forest strata position and niche shifts between juvenile and adult spiders

**DOI:** 10.3897/BDJ.13.e171693

**Published:** 2025-11-03

**Authors:** Ricardo Costa, Pedro Cardoso, François Rigal, Paulo A. V. Borges

**Affiliations:** 1 University of Azores, CE3C—Centre for Ecology, Evolution and Environmental Changes, Azorean Biodiversity Group, CHANGE —Global Change and Sustainability Institute, School of Agricultural and Environmental Sciences, Rua Capitão João d’Ávila, Pico da Urze, 9700-042, Angra do Heroísmo, Azores, Portugal University of Azores, CE3C—Centre for Ecology, Evolution and Environmental Changes, Azorean Biodiversity Group, CHANGE —Global Change and Sustainability Institute, School of Agricultural and Environmental Sciences, Rua Capitão João d’Ávila, Pico da Urze, 9700-042 Angra do Heroísmo, Azores Portugal; 2 LIBRe – Laboratory for Integrative Biodiversity Research, Finnish Museum of Natural History, University of Helsinki, Helsinki, Finland LIBRe – Laboratory for Integrative Biodiversity Research, Finnish Museum of Natural History, University of Helsinki Helsinki Finland; 3 IUCN SSC Monitoring Specialist Group, Angra do Heroísmo, Azores, Portugal IUCN SSC Monitoring Specialist Group Angra do Heroísmo, Azores Portugal; 4 CE3C - Centre for Ecology, Evolution and Environmental Changes, CHANGE – Global Change and Sustainability Institute, Faculty of Sciences, University of Lisbon, Lisbon, Portugal CE3C - Centre for Ecology, Evolution and Environmental Changes, CHANGE – Global Change and Sustainability Institute, Faculty of Sciences, University of Lisbon Lisbon Portugal; 5 IUCN SSC Atlantic Islands Invertebrate Specialist Group, Angra do Heroísmo, Azores, Portugal IUCN SSC Atlantic Islands Invertebrate Specialist Group Angra do Heroísmo, Azores Portugal; 6 Environment and Microbiology Team, Université de Pau et des Pays de l’Amour, Pau Cedex 64013, France Environment and Microbiology Team, Université de Pau et des Pays de l’Amour Pau Cedex 64013 France

**Keywords:** islands, Macaronesia, laurissilva, traits, ontogeny

## Abstract

Functional trait analyses have become a vital part of ecological and evolutionary research in recent years. Nevertheless, this progress highlights the persistent and significant gaps in our knowledge of species traits, a limitation known as the Raunkiæran shortfall. For spiders, the difficulty in properly identifying immature specimens has often contributed to discarding the contribution of these lifestages to intraspecific functional variability and community structure. Species microhabitat preferences along the vertical gradient in forest biomes are amongst the traits frequently unknown for spider juveniles, despite their relevance for multiple aspects of spiders' ecology. To bridge this knowledge gap, in this study, we used spider community data collected from the native forests on two islands belonging to the Azores Archipelago, a well-characterized and species-poor system ideal for trait-focused studies. Our goals were to compare the mean verticality and vertical range of adult and juvenile spiders belonging to different hunting guilds (hunters and web weavers) and ballooning propensity (frequent, occasional and rare ballooners). We did this using two-sample paired Wilcoxon signed rank tests and Kruskal–Wallis tests, followed by Dunn’s tests to check for differences in the variation of adult and juvenile verticality from species belonging to different functional groups. Across 22 species sampled at 16 sites on two islands, adult and juvenile spiders did not differ in their mean vertical position within the forest strata. Unexpectedly, however, adults occupied a significantly broader vertical range than juveniles, indicating greater habitat flexibility than anticipated. The pattern observed for vertical range remained when looking at the two hunting guilds considered (hunters and web-weavers), although, in the case of hunters, adults tended to have lower mean verticality than juveniles. Finally, for the three categories of ballooning propensity (frequent, occasional and rare), we observed that juveniles of rare ballooners had higher mean verticality, while, for all categories, these tended to have lower values of vertical range. Our findings show the importance of including juveniles in microhabitat studies, as well as how this seems to vary across functional groups. It is hoped that this study will serve as a valuable baseline to future research aiming to better incorporate immature life stages in spider community ecology, particularly as new standardised methodologies are developed to reliably associate juveniles with their adult forms.

## Introduction

Functional diversity analyses are a growing component of ecological and evolutionary research that focuses on characterising biodiversity based on functional traits (Hortal et al. 2015), a trait, in this case, being considered a phenotypic attribute that influences an organism's fitness in an ecosystem (Violle et al. 2007). The lack of knowledge or gaps that exist in species traits and their functions is known as the Raunkiæran shortfall (Hortal et al. 2015). This gap can be caused by other shortfalls, for example, the lack of species knowledge (i.e. Linnean shortfall) will render it impossible to obtain further ecological and evolutionary information (Hortal et al. 2015), but also by not proper standardisation of trait gathering that does not account for its variability (e.g. at the intraspecific level) (de Bello et al. 2025). Multiple aspects can influence trait variability, which depend on the level or scale at which the trait is being obtained (e.g. species-level or population-level) (Hortal et al. 2015), but also genetic variation, plasticity, phenological stage and ontogenetic development (de Bello et al. 2025).

When working with small, often inconspicuous, organisms such as spiders, classical species delimitation tends to be based on the morphology of the male and female copulatory structures. As these structures are only visible after they moult into the adult stage, this leads to most ecological studies focusing only on adult spider specimens (e.g. Coddington et al. (1996), Dobyns (1997)). Although DNA-based methods can facilitate the identification of specimens, including immature stages (e.g. Meiklejohn et al. (2013), Emerson et al. (2016)), they still require a properly validated reference database (Ratnasingham and Hebert 2007, Robinson et al. 2009), as well as considerable time and financial resources, making them impractical for large sample sizes. Although metabarcoding has been suggested as a tool to target this issue, quantifying the presence of taxa in bulk samples still varies greatly between groups (see Domènech et al. (2022)) and does not enable the association of species with their traits. Furthermore, small specimens might require the destruction of the entire body (Crespo et al. 2018) for DNA extraction, leading to loss of primary data.

Discarding immature specimens might overlook their unique contribution from different ontogenetic stages to the species' functional variability (Sanders et al. 2015, Yu et al. 2021). Furthermore, as juvenile spiders often make up the majority of specimens sampled (Wise 1993, Coddington et al. 1996, Emerson et al. 2016, Domènech et al. 2022), the inclusion of these lifestages becomes necessary for studies that aim to have a more complete view of each species' contribution to macroecological patterns (Domènech et al. 2022).

Vertical microhabitat preferences are a trait of particular importance in forest systems, as the microclimatic and vegetation structure gradients might influence the vertical distribution of spiders along the ground-canopy gradient (Lapinski and Tschapka 2018, Xing et al. 2023). Verticality changes according to different aspects of spiders' ecology (Foelix 2025), such as hunting strategy (Cardoso et al. 2011, Domènech et al. 2021), feeding preferences (Blackledge et al. 2003, Sanders et al. 2015) and dispersal ability (e.g. Sorensen (2003), Domènech et al. (2021)). For example, species that hunt using webs might be more dependent on the vegetation structure and so might be more bound to a specific microhabitat above ground-level (Rypstra et al. 1999, Amaral Nogueira and Pinto-da-Rocha 2016). Meanwhile, species that balloon might be more prone to inhabit arboreal microhabitats. Ballooning is thought to be one of the main ways for spiders to disperse over long distances (Thomas 1996, Szymkowiak et al. 2007, Gillespie et al. 2012). This usually depends on wind speed and electrostatic forces (Montes and Gleiser 2024) and, in forest systems, it is thought to happen closer to the canopy top (Bell et al. 2005, Montes and Gleiser 2024). Recent literature on ballooning has shown that even large spiders from groups that do not often balloon, such as large ground dwellers, might do so as juveniles (Buzatto et al. 2021, Rossi et al. 2021, Guerra et al. 2025). However, these might need to move to suitable microhabitats to do so, which might lead to juveniles having to climb vegetation (Rossi et al. 2021) and so might colonize microhabitats largely different from those of the adults. Despite the difficulty in identifying juveniles, some studies have tried to include the microhabitat preference of spiderlings in their research questions (e.g. Fasola and Mogavero (1995), Lapinski and Tschapka (2018), Campuzano et al. (2019), Niedobová et al. (2024)). However, in most cases, these studies focus on very few species (e.g. Lapinski and Tschapka (2018), Campuzano et al. (2019)), leading to most communities remaining poorly studied (but see Fasola and Mogavero (1995)).

One way to obtain better insights into juvenile spider ecology is to study well-known, simpler systems. For this purpose, island systems can be particularly suitable, mostly since these can act like natural laboratories helping us to understand more complex settings (Whittaker et al. 2017). The Azores Archipelago is a good example, as it is a set of small, young and isolated islands (Gaspar et al. 2011, Triantis et al. 2011). Many studies over the last 30 years have accumulated a great amount of knowledge of species identity, distribution, conservation status and functional ecology (Gaspar et al. 2011, Rigal et al. 2017, Macías-Hernández et al. 2020, Borges et al. 2022, Lhoumeau and Borges 2023, Oyarzabal et al. 2024, Oyarzabal et al. 2025).

In our study, we used data obtained from adult and juvenile spiders collected from the most pristine native forest fragments of the Azores to compare their verticality patterns (see Malumbres-Olarte et al. (2019)). Our goals were to compare island adult and juvenile spiders belonging to different hunting guilds (hunters and web weavers) and ballooning propensity (frequent, occasional and rare ballooners). Our three hypotheses were: i) juveniles have the same mean verticality, but larger vertical range than their adults as they tend to search for better places to disperse; ii) this divergence will be more strongly seen in juvenile hunters and not web-weavers, as the latter should already live in arboreal habitats that favour ballooning; iii) juveniles of rare ballooners will diverge from adults contrary to occasional and frequent ballooners in mean verticality and vertical range.

## Material and methods

### Study system

The Azores Archipelago consists of nine volcanic oceanic islands arranged in three groups (western, central and eastern) (Fig. 1a, b) between ~ 36.9–39.7° N and 31.3–25.0° W. Lying ~ 1,600 km west of mainland Portugal and ~ 3,900 km east of eastern North America, the Archipelago sits near the Azores Triple Junction, where the North American, Eurasian and Nubian (African) plates interact, producing active tectonism and a diverse volcanic geomorphology (calderas, stratovolcanoes, rift zones, geothermal fields). This isolation and geodynamic setting underpin the region’s distinctive biota and notable levels of endemism (Borges et al. 2022).

All islands have a temperate oceanic climate with high humidity; Terceira, for example, having around 2800 mm annual rainfall and temperatures varying between 6°C and 25°C (Henriques et al. 2016). Human activities during the last 500 years have restricted native vegetation to high elevations (above 500 m a.s.l.), with only about 5% of the original habitats persisting (Gaspar et al. 2011). The surviving forests are mostly dominated by *Juniperus brevifolia*, are very dense, hyper-humid and present a dense cover of bryophytes on all substrata (Elias et al. 2016). In this study, we used data collected from two islands (Pico and Terceira), both younger than 0.4 Ma (Hildenbrand et al. 2014), which include some of the most pristine fragments of native forest remaining in the Archipelago (Gaspar et al. 2011). Terceira is the island with three of the largest and most untouched native forests fragments in Azores (Serra de Santa Bárbara, Biscoito da Ferraria-Pico Alto and Terra-Brava), which occupy 2111 ha, making 5% of the area of the island (Fig. 1c) (Gaspar et al. 2008). Pico is the Azorean island of most recent origin and also the one with the highest elevation (2351 m); however, its remaining native forests are much more fragmented, occupying 2% of the 444 km^2^ of the island (Fig. 1d) (Gaspar et al. 2008).

### Sampling protocol and identification

Spiders were sampled in 50 x 50 m plots in native mesic forest sites dominated by endemic trees and shrubs, six in Pico and 10 in Terceira (see detailed location of sites at My Maps Terceira and Pico Sites) (Cicconardi et al. 2017, Borges et al. 2018a, Malumbres-Olarte et al. 2019, Malumbres‐Olarte et al. 2021). Plots in Terceira were selected, based on the presence of pristine forest within the framework of ISLAND-BIODIV project (Cicconardi et al. 2017, Borges et al. 2018a) and were sampled in 2012. For Pico, the sites were chosen within the framework of MACDIV project to study distance-decay between sites on patterns of alpha and beta diversity (Borges et al. 2018a, Malumbres‐Olarte et al. 2021) and were sampled in 2016. The chosen plots are now part of two long-term monitoring studies, the SLAM project (Lhoumeau and Borges 2023, Borges 2025) and the BioMonI project (Borges et al. 2025).

The sampling followed the COBRA (Conservation Oriented Biodiversity Rapid Assessment) sampling protocol (Cardoso 2009, Borges et al. 2018b). This protocol has been used to collect data to study diversity patterns at multiple spatial scales —island (Malumbres-Olarte et al. 2019, Malumbres-Olarte et al. 2020), archipelago (Malumbres‐Olarte et al. 2021, Costa et al. 2023), continental areas (Cardoso 2009, Domènech et al. 2021, Domènech et al. 2022) and even from different continents (Cardoso et al. 2011, Malumbres-Olarte et al. 2018). Its core version targets four different strata each with a specific sampling technique: ground-floor was sampled with pitfall traps; short/herbaceous vegetation with net sweeping, both during the day and night; trunks and foliage up to ca. 2 m high with nocturnal active aerial search; and canopy foliage with beating vegetation on to a sheet both during day and night to maximum of 3 m high (for more details see Cardoso (2009), Borges et al. (2018b)). Islands were sampled between June and September, targeting the period of maximum arthropod richness in these forests (Malumbres-Olarte et al. 2019). The spider species considered in this study were identified using morphological characters, adult specimens being confirmed through barcoding (mitochondrial cytochrome c oxidase 1) (for more details, see Malumbres-Olarte et al. (2019)). We then checked if species names were up to date on their nomenclature, using the 'checknames' function in the *arakno* R package (Cardoso and Pekár 2022). Only juvenile specimens from later instars, often close to becoming adults, were identified to species level by one of the authors (PAV Borges), based on his experience in the Azores Archipelago arachnofauna. Of the juveniles collected, those in very early instars were excluded: on Terceira Island, 197 out of 4,540 individuals (4.3%), and on Pico Island, 407 out of 3,085 individuals (13.2%). Species hunting guild (hunter or web-weaver) and dispersal propensity (frequent, occasional or rare ballooner) were obtained from Macías-Hernández et al. (2020). All voucher specimens are deposited at EDTP — Entomoteca Dalberto Teixeira Pombo, Campus de Angra do Heroísmo, Portugal and data can be accessed from Malumbres-Olarte et al. (2019).

### Obtaining verticality values

Following the approach described by Costa et al. (2023), we calculated two verticality metrics. These were: mean verticality (from hereon AVG V), which describes where the species most often occurs in the ground-canopy gradient; and standard deviation verticality (from hereon STD V), which is used as a proxy of vertical range. To obtain these values, we multiplied a score given to each sampling method (0 for pitfall, 1 for sweeping, 2 for active aerial search and 3 for beating) by the normalised relative abundance in that stratum (i.e. the proportion of each stratum for the species, maximising at 1). This way, higher values of mAVG V were given to species occupying higher strata (closer to the canopy). These values were then divided by 3 to obtain values bounded between 0 and 1 for mAVG V and 0 and 0.5 for mSTD V see Costa et al. (2023) for further details on the verticality calculation). Next, for each species in each plot, we obtained the AVG V and STD V values. The calculation was implemented separately for the juvenile and adult lifestage specimens.

### Statistical analysis

We applied Wilcoxon signed rank tests, as AVG A and STD V distributions did not fulfil the statistical assumptions for the t-test, to quantify whether both verticality values were significantly different between adults and juveniles of the same species in sites where both life stages co-occurred. This was then repeated separately for the hunting guilds (hunters and web-weavers) and ballooning propensity groups (frequent – F; occasional – O and rare – R). The difference in verticality values of AVG V (AVG diff) and STD V (STD diff) obtained for species in sites with both adult and juvenile specimens was compared using Kruskal–Wallis tests (KW), in order to check for significant variations between groups. When the KW was statistically significant at alpha < 0.05, Dunn’s tests were performed to identify statistically significant pairwise differences between hunting guilds and dispersal propensity groups. All statistical analyses were performed using the R software version v.4.4.0 (R.Core Team 2025).

## Results

### General patterns and species studied

From a dataset of 29 species sampled in Terceira and 32 in Pico, we selected a total of 17 species for Terceira (2292 adults and 3133 juveniles) and 20 for Pico (1219 adults and 1486 juveniles). This included both indigenous and introduced species (Borges et al. 2022), which had both lifestages sampled in the same site (Table 1). The selected species belonged to a total of 12 different families, with Linyphiidae being the most species-rich (eight taxa). Hunters were more diverse than Web-weavers (13 vs. 9, respectively), while rare ballooners were less diverse (five species) vs. frequent and occasional (nine and eight species, respectively). Verticality varied between 0 and 1 for AVG V for both adults and juveniles, while, for STD V, adult spiders varied between 0 and 0.416 and juveniles between 0 and 0.392 (see Suppl. material 1).

### Comparison between adult and juvenile spiders

**Figure 2.**
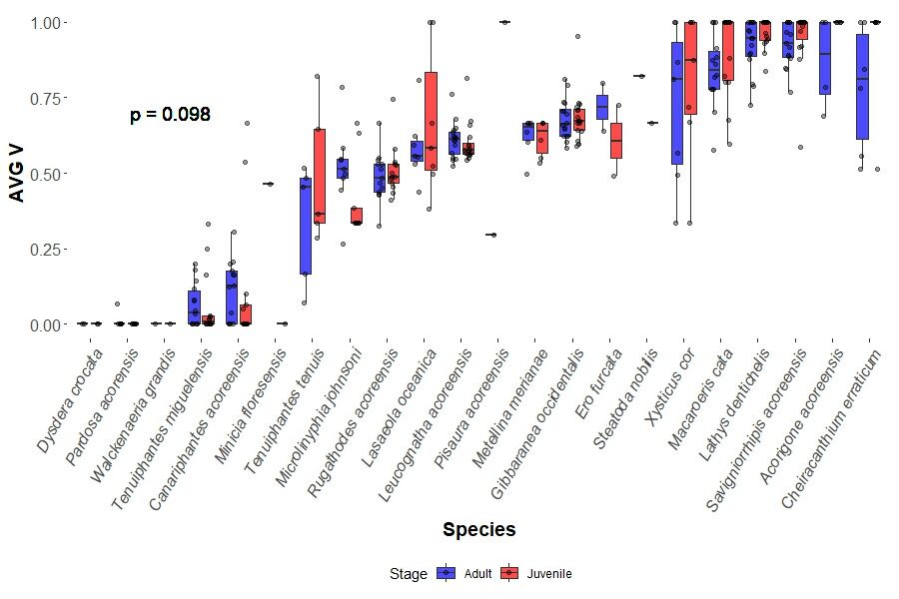
Species boxplots distinguish values obtained for adults and juveniles, each dot representing a species in a site for AVG V values. The p-value represents the significance obtained between the studied groups by the KW analysis.

**Figure 3.**
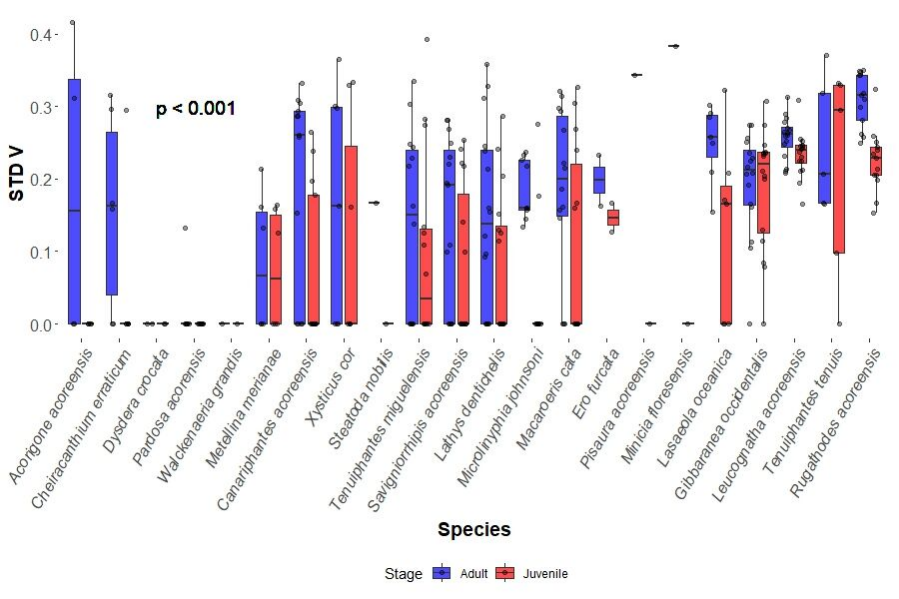
Species boxplots distinguish values obtained for adults and juveniles, each dot representing a species in a site for STD V values. The p-value represents the significance obtained between the studied groups by the KW analysis.

As predicted, adult spiders in the studied sites did not diverge in their median AVG V (0.616 for adults interquartile range = 0.388, from hereon IQR) from their respective juveniles (0.635 for adults IQR = 0.388) in the site (Fig. 2). However, contrary to predictions, juveniles did not show higher median values of STD V (0.104, IQR = 0.231), but significantly lower ones instead (p < 0.001) when compared to adults in the same site (0.214, IQR = 0.184) (Fig. 3). Indeed, species in each site tended to show little change in AVG V values (median change = 0, IQR = 0.107), but a tendency of positive values of STD V change between juveniles and adults (median change = 0.027, IQR = 0.114).

### Hunting guilds' verticality variation

**Figure 4.**
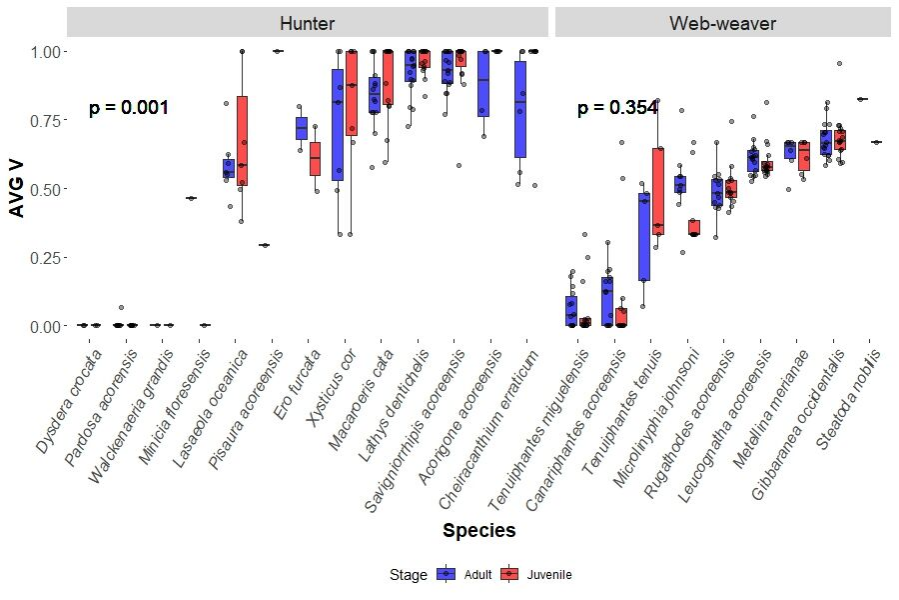
Species boxplots distinguish values obtained for adults and juveniles, each dot representing a species in a site. This shows the species sampled AVG V values divided by hunting guild (hunters and web-weavers). The p value represents the significance obtained between the studied groups by the KW analysis.

**Figure 5.**
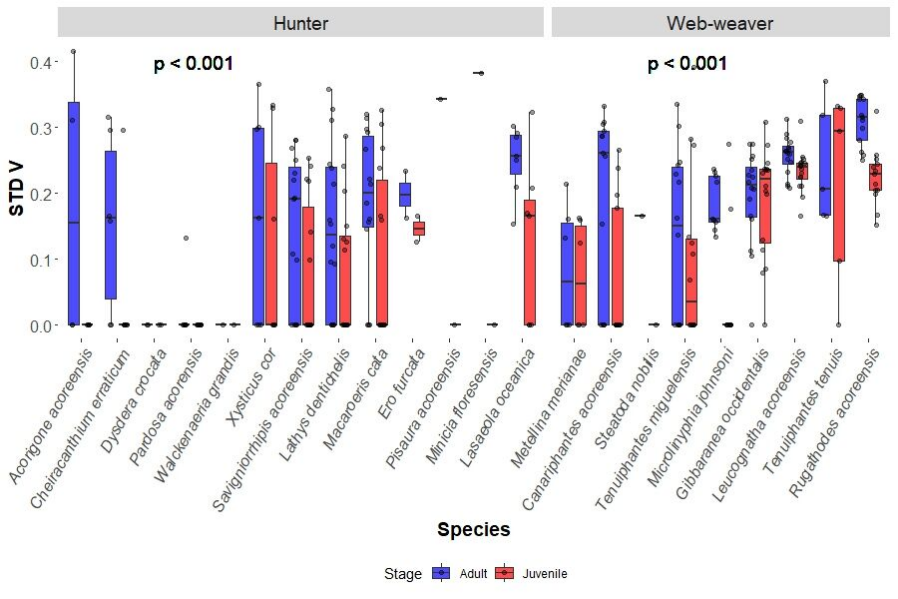
Species boxplots distinguish values obtained for adults and juveniles, each dot representing a species in a site. This shows the species sampled STD V values divided by hunting guild (hunters and web-weavers). The p value represents the significance obtained between the studied groups by the KW analysis.

Regarding hunting guilds, hunter juveniles showed significantly larger values of AVG V (0.985, IQR = 0.333) than the adults (0.844, IQR = 0.398) on each site (p = 0.001). Meanwhile, web-weaver juveniles AVG V (0.535, IQR = 0.396) did not significantly differ from their respective adults (0.524, IQR = 0.447) in each site (Fig. 4). As for STD V, hunter juveniles showed significantly lower values of STD V (0, IQR = 0.161) than the adults (0.161, IQR = 0.275) on each site (p < 0.001). The same result was obtained for web-weavers, with juveniles having lower median STD V (0.195, IQR = 0.239) than the adults (0.232, IQR = 0.117) (Fig. 5).

Regarding the difference in AVG V (AVG V diff) between adults and juveniles of hunters and web-weavers, the first had significantly lower values (p = 0.003; median = 0, mean = -0.051, IQR = 0.114 for hunters and median = 0, mean = 0.003, IQR = 0.047 for web-weavers) (see Suppl. material 2). When looking at the difference in STD V (STD V diff) hunters and web-weavers, there were no significant differences (median = 0.002, mean = 0.080, IQR = 0.136 for hunters and median = 0.033, mean = 0.057, IQR = 0.105 for web-weavers) (see Suppl. material 3).

### Ballooning propensity groups verticality variations

**Figure 6.**
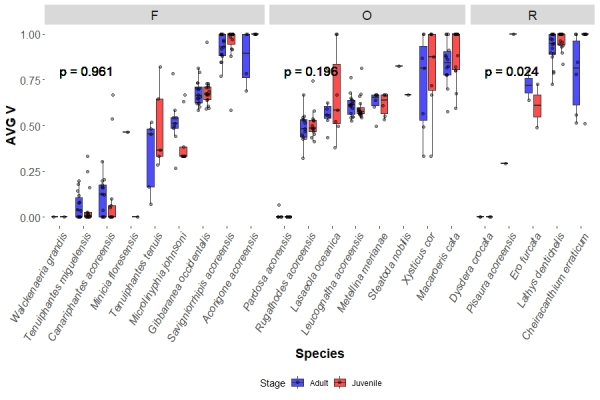
Species boxplots distinguish values obtained for adults and juveniles, each dot representing a species in a site. This shows the species sampled AVG V values divided by ballooning propensity (frequent – F, occasional – O and rare – R). The p value represents the significance obtained between the studied groups by the KW analysis.

**Figure 7.**
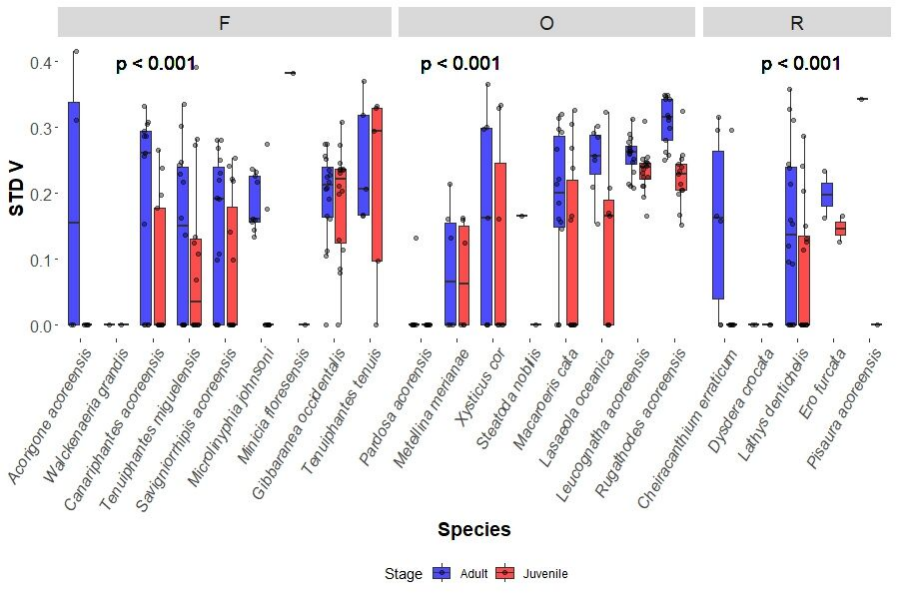
Species boxplots distinguish values obtained for adults and juveniles, each dot representing a species in a site. This shows the species sampled STD V values divided by ballooning propensity (frequent – F, occasional – O and rare – R). The p value represents the significance obtained between the studied groups by the KW analysis.

Regarding ballooning propensity categories, in the case of frequent and occasional ballooners, juveniles showed no difference in AVG V (0.586, IQR = 0.774 and 0.583, IQR = 0.289, frequent and occasional, respectively) from the adults (0.531, IQR = 0.651 and 0.601, IQR = 0.265, frequent and occasional, respectively). Rare ballooner juveniles AVG V (1, IQR = 0.086), however, showed significantly higher values than their respective adults (0.893, IQR = 0.233) in each site (p = 0.024) (Fig. 6). As for STD V, for all ballooning categories, juveniles had significantly lower (for all p < 0.001) values (frequent: 0, IQR = 0.221; occasional: 0.167, IQR = 0.240, rare: 0, IQR = 0.129) than their respective adults (frequent: 0.206, IQR = 0.156; occasional: 0.250, IQR = 0.141, rare: 0.158, IQR = 0.242) in each site (Fig. 7).

Regarding the difference in AVG V between adult and juvenile, neither of the ballooning propensity groups significantly diverged from each other (median = 0, mean = -0.003, IQR = 0.127 for frequent ballooners, median = 0, mean = -0.024, IQR = 0.088 for occasional and median = 0, mean = -0.075, IQR = 0.084 for rare ones) (see Suppl. material 4). When looking at the difference in STD V of ballooning categories, there were no significant differences between the groups (median = 0.030, mean = 0.072, IQR = 0.138 for frequent ballooners, median = 0.022, mean = 0.058, IQR = 0.103 for occasional and median = 0.040, mean = 0.080, IQR = 0.102 for rare) (see Suppl. material 5).

## Discussion

The juvenile stage of spiders makes up much of the life span of most species and often comprises the majority of individuals in community-wide studies (Wise 1993, Coddington et al. 1996, Emerson et al. 2016, Domènech et al. 2022). However, the struggle to sample and properly identify them makes gathering ecological information about this portion of spiders' life stages a challenging task (Coddington et al. 1996, Dobyns 1997). In our study, we used a well-studied species-poor island system (two Azorean Islands) to try and gain some insight into the differences in vertical microhabitat preferences between adults and juveniles along the ground-canopy gradient of native forests. We show that these differences do occur and seem to have a relationship with some functional aspects of spider ecology (hunting strategy and ballooning propensity).

### Verticality variation between adult and juvenile spiders

Unlike holometabolous insects, whose larval stages often have drastically different lifestyles when compared to adults (Wiegmann et al. 2009), spiders undergo direct development in which hatchlings resemble miniature adults and maintain a similar body plan throughout their juvenile and adult phases (Foelix 2025). This might lead to only slight changes in their functional ecology as they grow, which, in the topic of this study, hypothetically would translate to similar verticality to that of the adults. Our overall results for mean verticality (AVG V) support this, with no differences being generally found between adult and juvenile spiders. This result matches those of previous studies, in which adults and especially later instars of juveniles have very similar vertical microhabitat preferences (Fasola and Mogavero 1995, Lapinski and Tschapka 2018, Niedobová et al. 2024). However, to our knowledge, this is the first study to take a species-focused community approach to determine vertical patterns observed between adult and juvenile spiders over multiple forest strata.

There are, however, aspects of spiders' life-history known to greatly differ between the two lifestages. One good example is regarding dispersal propensity, as the juvenile is known to be an important dispersal stage in many spider groups (Blandenier 2009, Buzatto et al. 2021, Rossi et al. 2021). Examples of this are known for many spider families from both mygalomorph, such as Actinopodidae, Atypidae and Halonoproctidae (Buzatto et al. 2021) and araenomorpha, such as Araneidae, Lycosidae, Theridiidae, amongst others (Blandenier 2009). With this in mind, one would assume that juveniles of most species would likely occur over a much larger range of microhabitats than their respective adults. However, contrary to our expectations, we observed the inverse tendency of change in vertical range, with adults tending to have larger values of standard deviation verticality (STD V) than juveniles. Juveniles from many spider species may be prone to, indeed, dispersing less, especially in an island setting (Malumbres‐Olarte et al. 2021), as it might lead to less survivability (Takada and Miyashita 2014). Other factors that might be influencing the pattern we obtained might include the presence of male specimens in adult samples. For many species, these are known to also move around to find a mate (e.g. Fasola and Mogavero (1995)), which might influence the vertical range in which we recorded adult spiders. Although a sex-based analysis is beyond the scope of this study, it is important to consider that, in time-restricted protocols, ontogeny can have an effect on the sampled community, as it can vary greatly between species (Fasola and Mogavero 1995).

It is also important to note that, by limiting species‐level identifications to juvenile late instars, individuals whose morphology closely matches adults, we excluded only a small fraction of our samples: 197 of 4,540 specimens on Terceira (4.3%) and 407 of 3,085 on Pico (13.2%). Such low exclusion rates fall well within accepted standards for arachnological surveys and indicate that our results remain broadly representative of both species richness and juvenile abundance. However, the fact that size makes juvenile spiders also harder to spot than adults by more active sampling methods (such as AAS - night Active Aerial Search) might have influenced the obtained values of STD V. Furthermore, our limit of 3 m of canopy height might have played a factor in this. As the canopy in some sites continues further up, we might obtain lower STD V for more arboreal species. However, investigating the recently available SLAM traps dataset from Lhoumeau et al. (2025), after controlling for site‐to‐site heterogeneity, there remains no evidence that total juvenile mean abundance differs amongst Ground, Understorey and Canopy strata. This matches the results obtained in the present study, as the strata sampled covered the entire canopy layer of the native forests. Therefore, there is a need to develop tools to properly overcome the complications of studying these lifestages, as they show us we might be missing more accurate information on the ecology of spiderlings (e.g. Emerson et al. (2016), Domènech et al. (2022)).

### Hunters and web-weavers

Juveniles of species classified as hunters exhibited higher AVG V values than their adult counterparts. In contrast, web-weavers tended to occasionally show positive shifts in mean vertically or no shift at all. Although this could point to hunters moving to higher strata in the canopy where dispersal is facilitated, this might not be the only factor, as, in general, hunters had much larger mean verticality. However, ballooning might not be the only reason behind the difference between the verticality patterns of adult and juvenile spiders, as these might also relate to the different hunting strategies implemented by each group. Web-weavers tend to depend more heavily on vegetation structure than hunters (Rypstra et al. 1999, Amaral Nogueira and Pinto-da-Rocha 2016). Due to this, juveniles might still depend on the same microhabitats along the vertical gradient to build their webs, which leads them to occupy similar vertical positions as adults (Fasola and Mogavero 1995, Takada and Miyashita 2014). Furthermore, by mostly catching prey using their webs, juvenile web-weavers might be less susceptible to predation when compared to those of hunter species (Takada and Miyashita 2014), although competition for web sites can still occur (Reitz and Trumble 2002). Meanwhile, hunter juveniles might be more vulnerable to predation by larger spiders while foraging amongst the vegetation, which could also favour their occupation of a different stratum than the adults (Lapinski and Tschapka 2018). Indeed, juveniles may look for more complex micro-habitats, since it has been shown that survival is related to habitat complexity, predatory arthropods suffering much higher mortality when hunting in simple, two-dimensional vegetation (Finke and Denno 2002, Takada and Miyashita 2014). However, the larger STD V of the adults of some hunters might also relate to the larger mean verticality of juveniles, as these might go further up than the sampled range. However, a preliminary analysis of the dataset of Lhoumeau et al. (2025) did not point to a clear disproportional increase in the mean verticality of the juveniles of these more arboreal hunters. It is important to note that not all hunters presented similar changes, with ground-dwelling species, such as *Dysdera crocata* C. L. Koch, 1838 and *Pardosa acorensis* Simon, 1883, showing no differences between adults and juveniles. In fact, the greatest differences both in mean verticality and vertical range seem to have happened between vegetation dwellers, mostly arboreal hunters, such as *Pisaura acoreensis* Wunderlich, 1992, *Acorigone acoreensis* (Wunderlich, 1992) or *Cheiracanthium erraticum* (Walckenaer, 1802). This once again points to the importance of different microhabitats when trying to understand the ecology of these small organisms.

### Ballooning categories

Studies on ballooning have pointed out that a major component of the ballooning community of spiders consists, in fact, of juveniles (Blandenier 2009, Buzatto et al. 2021). It is no wonder then that more and more cases are being reported from species for which adults would be too large to disperse in this way, such as for many mygalomorphs (Rossi et al. 2021, Buzatto et al. 2021). Our results partly match this scenario. The best ballooners tend to be web-weaving spiders, which, in our study, did not differ between adults and juveniles at the same site. This result is also confirmed by rare ballooners being the only ones that showed significantly larger values of AVG V for the juveniles. In fact, these were the functional groups that showed the largest variation in AVG V between adults and juveniles, which strengthens how important dispersal seems to be connected with the presence in higher vertical strata (Bell et al. 2005, Montes and Gleiser 2024), at least in Azores native forests. It is worth mentioning that the classification we used for ballooning is family-level based and so, the species-specific and lifestage ballooning propensity for the studied taxa is unknown. However, rare ballooners also included fewer species (5) from which three are introduced to the Azores and so it is unknown how well adapted these are to the native forest system (but see Lhoumeau and Borges (2023)). Additionally, although many of the juveniles tended to be more arboreal than their adults in this group, the reason can be species-specific due to other ecological factors not considered in our study, such as preferred prey by different instars (Foelix 2025).

## Conclusion

Overall, as predicted, juvenile spiders tend to maintain the same values of mean verticality, although this seems to vary between functional groups. Contrary to our predictions, juveniles showed lower vertical range than their respective adults. These findings shed light on the importance of considering different ecological components of species in a community in order to understand the importance of verticality for spiders from different lifestages. We consider our findings, which were obtained from a limited group of species occurring in small patches of native forests, to be a baseline for future studies that assess more complex communities from other islands and continental systems. Future works should focus not only on continuing to include juveniles in analyses (possible using molecular methods; Emerson et al. (2016), Emerson et al. (2022)), but also try to obtain accurate species-based information.

## Supplementary Material

911718CF-C87A-52A3-9002-1B2C71BD21D8 10.3897/BDJ.13.e171693.suppl1 Supplementary material 1 Appendix 1 Data type Table Brief description Verticality values (AVG V and STD V) obtained for adult and juvenile spiders at each site for each studied species. File: oo_1396055.xlsx https://binary.pensoft.net/file/1396055 Ricardo Costa

4C0DF588-2AC0-5B53-AD12-A432B612D307 10.3897/BDJ.13.e171693.suppl2 Supplementary material 2 Appendix 2 Data type Figure Brief description Boxplots and violin plots showing the difference in AVG V values (AVG diff) between adult and respective juvenile spiders in each site obtained for each hunting guild (hunter and web-weaver). The p value represents the significance obtained between the studied groups by the KW analysis. File: oo_1396211.png https://binary.pensoft.net/file/1396211 Ricardo Costa

F805BE7D-4085-5001-A1A4-4DAD61D22197 10.3897/BDJ.13.e171693.suppl3 Supplementary material 3 Appendix 3 Data type Figure Brief description Boxplots and violin plots showing the difference in STD V values (STD V diff) between adult and respective juvenile spiders in each site obtained for each hunting guild (hunter and web-weaver). The p value represents the significance obtained between the studied groups by the KW analysis. File: oo_1396212.png https://binary.pensoft.net/file/1396212 Ricardo Costa

FEF2E393-A99E-53BA-A19A-D90B6DE3DC44 10.3897/BDJ.13.e171693.suppl4 Supplementary material 4 Appendix 4 Data type Figure Brief description Boxplots and violin plots showing the difference in AVG V values (AVG V diff) between adult and respective juvenile spiders in each site obtained for each ballooning propensity category (F – frequent, O – occasional and R – rare). The p value represents the significance obtained between the studied groups by the KW analysis. File: oo_1396230.png https://binary.pensoft.net/file/1396230 Ricardo Costa

8D38DD4D-A9E8-5069-9E78-77612D1CD340 10.3897/BDJ.13.e171693.suppl5 Supplementary material 5 Appendix 5 Data type Figure Brief description Boxplots and violin plots showing the difference in STD V values (STD V diff) between adult and respective juvenile spiders in each site obtained for each ballooning propensity category (F – frequent, O – occasional and R – rare). The p value represents the significance obtained between the studied groups by the KW analysis. File: oo_1396232.png https://binary.pensoft.net/file/1396232 Ricardo Costa

## Figures and Tables

**Figure 1a.**
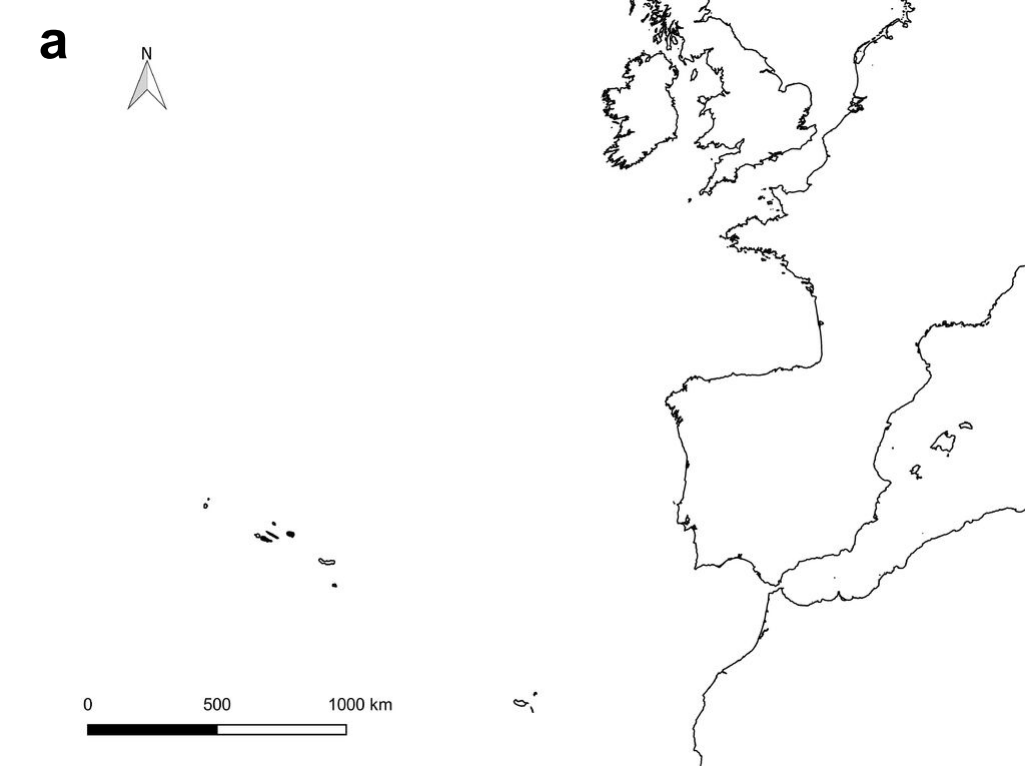


**Figure 1b.**
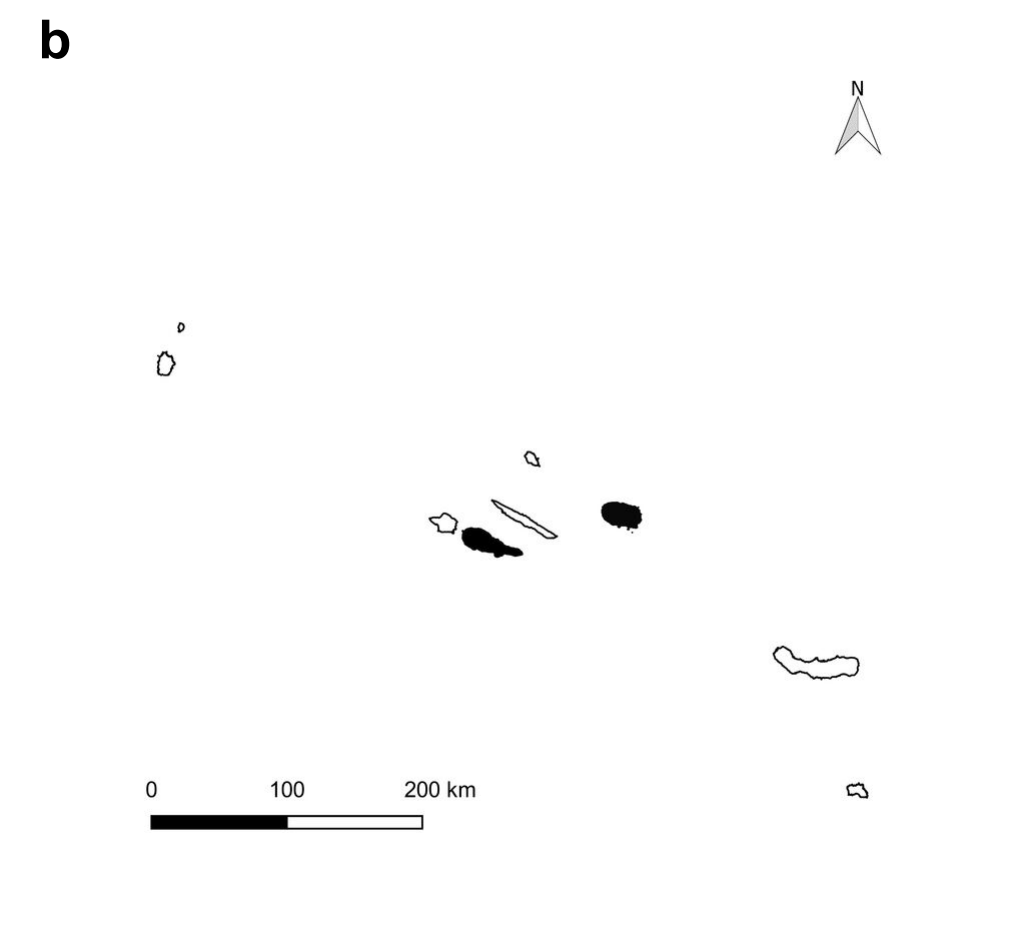


**Figure 1c.**
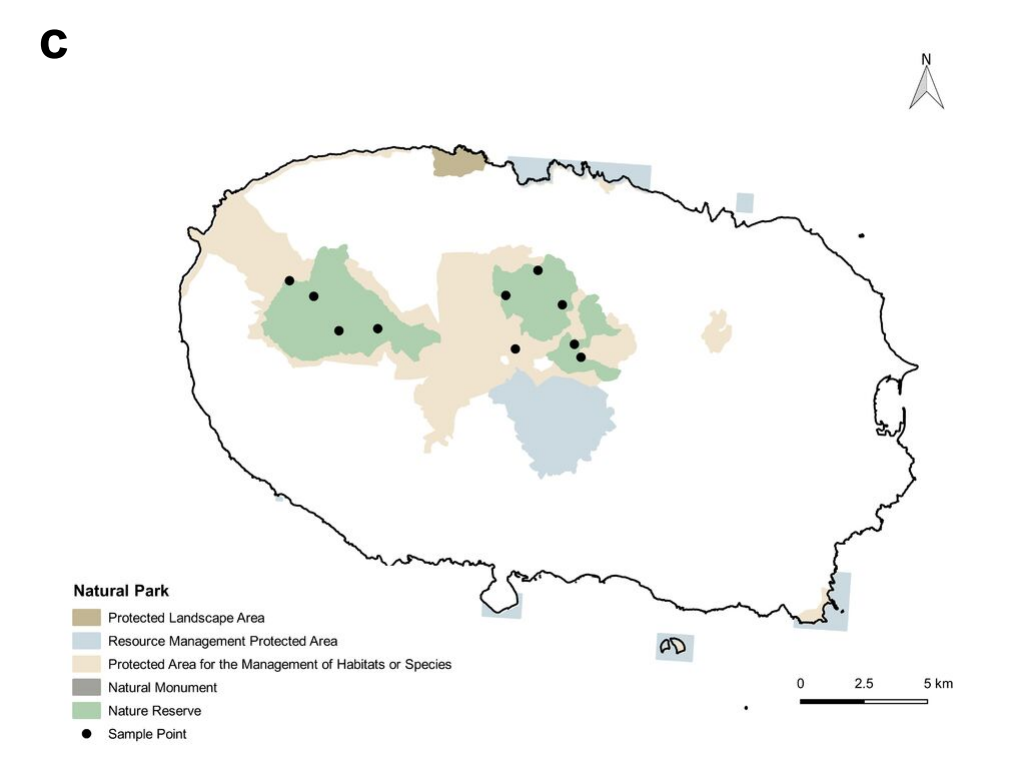


**Figure 1d.**
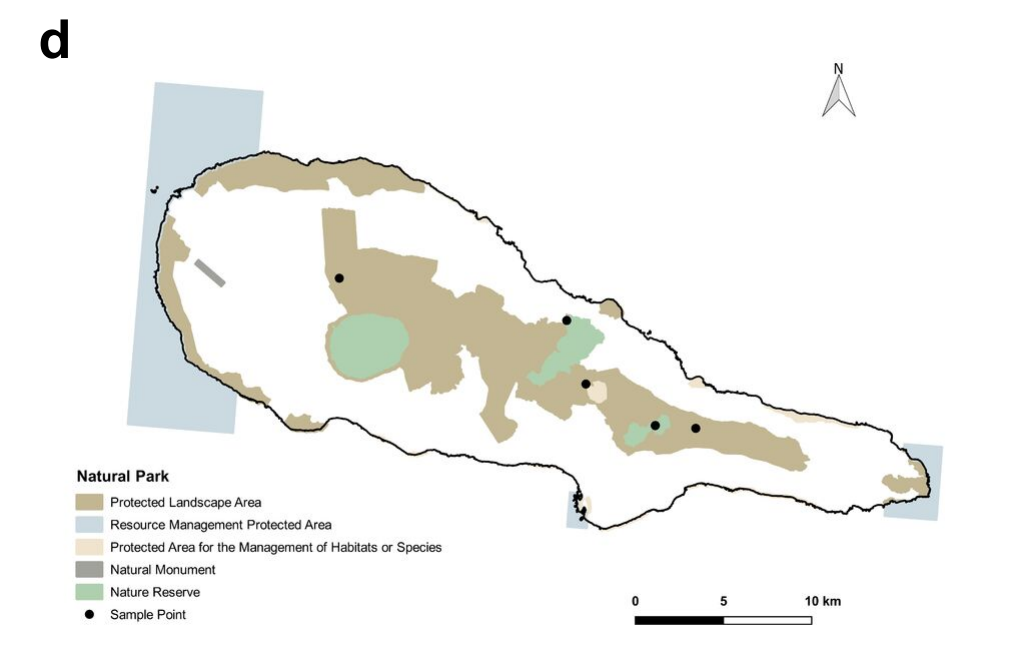


**Table 1.** Number of sites and abundance values of adult and juvenile Azores spider species used in the analysis from each island together with relevant ecological information: Dispersal – different ballooning propensity categories (F - frequent ballooners, O - occasional ballooners, R- rare ballooners); Hunting strategy – hunter for species that do not build a web to capture prey and web-weavers for those that do build one; Colonisation origin – species classification as endemic, native non-endemic and introduced to the Azores (following Borges et al. (2022)).

Species	Dispersal	Hunting strategy	Colonisation origin	Number of sites	Terceira Adults	Terceira Juveniles	Pico Adults	Pico Juveniles
*Acorigone acoreensis*	F	Hunter	Endemic	4	38	5	14	1
*Canariphantes acoreensis*	F	Web-weaver	Endemic	13	88	81	28	14
*Cheiracanthium erraticum*	R	Hunter	Introduced	6	21	24	2	1
*Dysdera crocata*	R	Hunter	Introduced	2	13	1	4	5
*Ero furcata*	R	Hunter	Introduced	2	20	8	NA	NA
*Gibbaranea occidentalis*	F	Web-weaver	Endemic	16	324	763	61	92
*Lasaeola oceanica*	O	Hunter	Endemic	7	56	18	22	4
*Lathys dentichelis*	R	Hunter	Native	16	167	371	72	131
*Leucognatha acoreensis*	O	Web-weaver	Endemic	16	330	426	157	228
*Macaroeris cata*	O	Hunter	Native	14	80	70	38	39
*Metellina merianae*	O	Web-weaver	Introduced	6	NA	NA	83	96
*Microlinyphia johnsoni*	F	Web-weaver	Native	9	90	8	52	66
*Minicia floresensis*	F	Hunter	Endemic	1	NA	NA	28	1
*Pardosa acorensis*	O	Hunter	Endemic	7	46	79	61	11
*Pisaura acoreensis*	R	Hunter	Endemic	1	23	2	NA	NA
*Rugathodes acoreensis*	O	Web-weaver	Endemic	13	536	263	176	166
*Savigniorrhipis acoreensis*	F	Hunter	Endemic	15	276	841	83	196
*Steatoda nobilis*	O	Web-weaver	Introduced	1	NA	NA	3	1
*Tenuiphantes miguelensis*	F	Web-weaver	Native	14	174	167	275	307
*Tenuiphantes tenuis*	F	Web-weaver	Introduced	5	NA	NA	23	36
*Walckenaeria grandis*	F	Hunter	Endemic	1	NA	NA	16	4
*Xysticus cor*	O	Hunter	Native	7	10	6	21	87
