# Supplementary figures and images for: Vertical forest strata position and niche shifts between juvenile and adult spiders
Source: Biodivers Data J. 2025 Nov 3;13:e171693. doi: 10.3897/BDJ.13.e171693 (PMC12603644; doi:10.3897/BDJ.13.e171693)

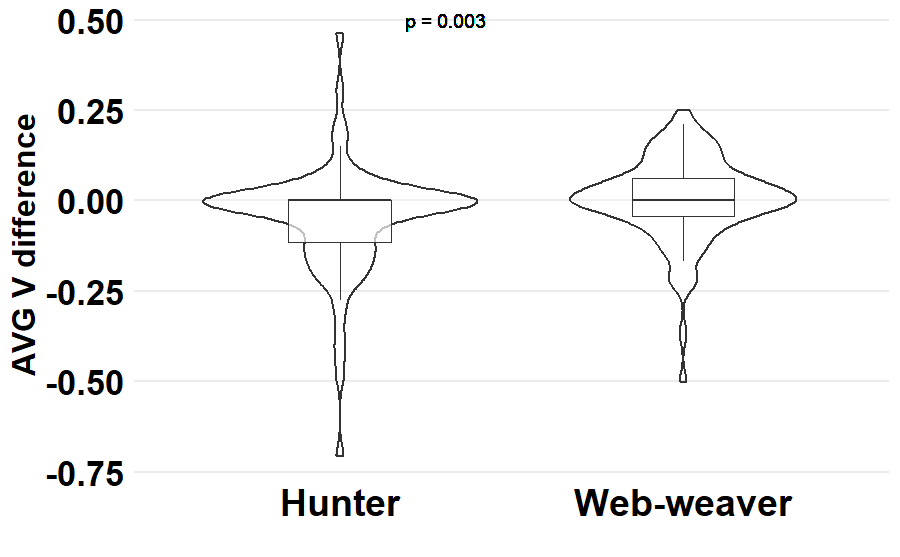

Supplement: Supplementary material 2 — Appendix 2 [file bdj-13-e171693-s002.png]

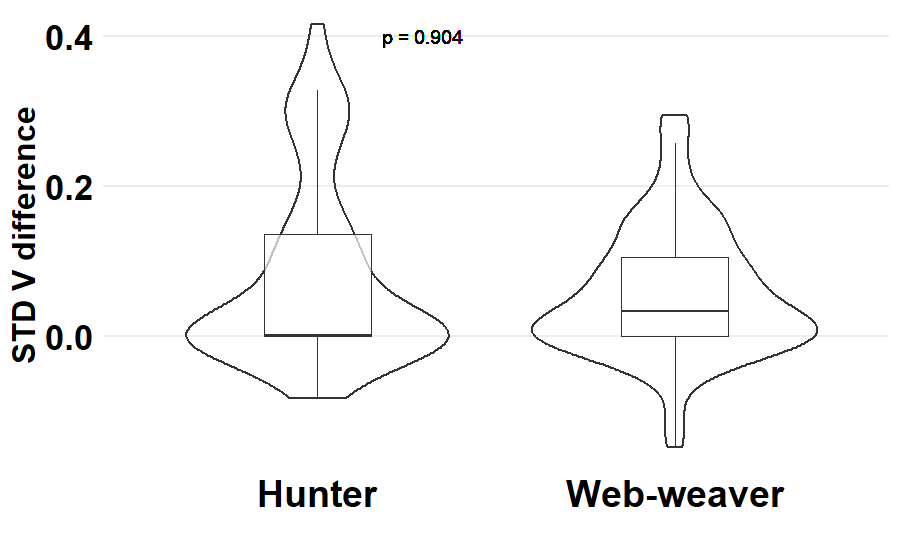

Supplement: Supplementary material 3 — Appendix 3 [file bdj-13-e171693-s003.png]

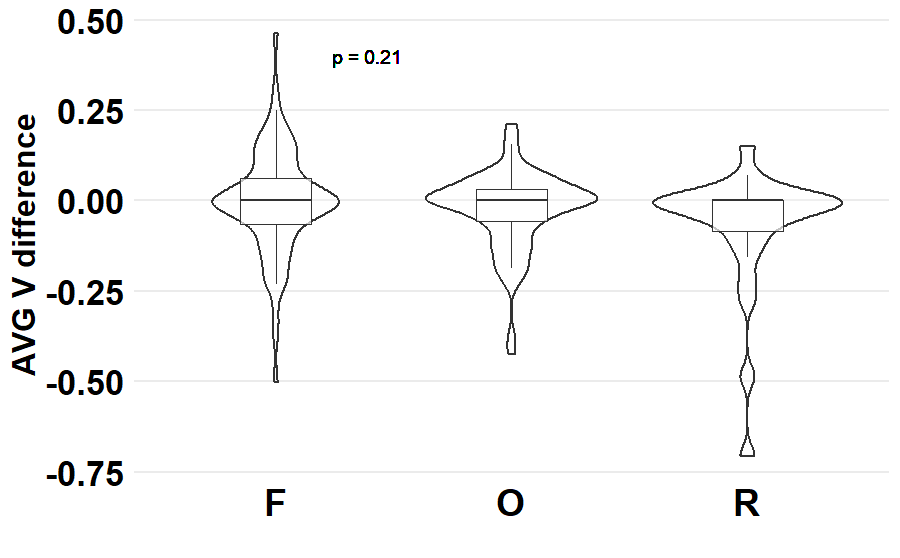

Supplement: Supplementary material 4 — Appendix 4 [file bdj-13-e171693-s004.png]

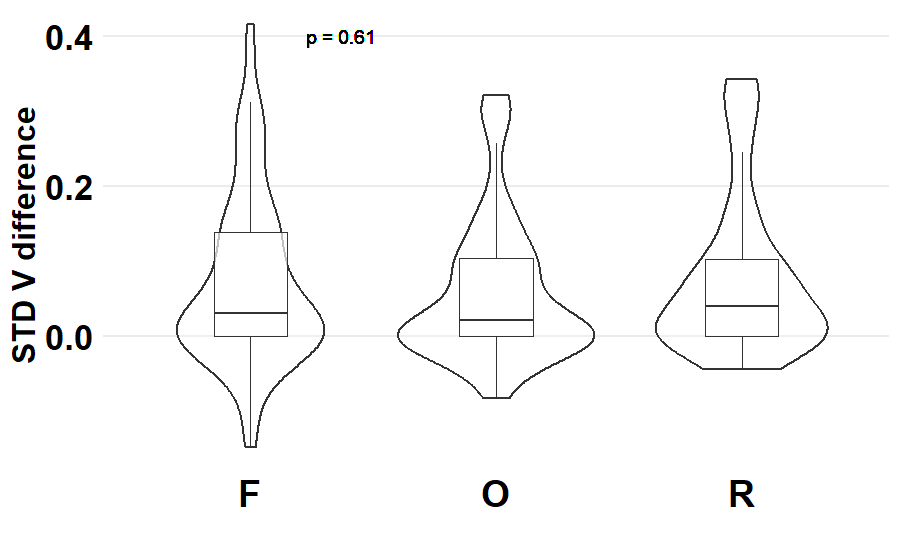

Supplement: Supplementary material 5 — Appendix 5 [file bdj-13-e171693-s005.png]
